# The Effect of Probiotics on Quality of Life, Depression and Anxiety in Patients with Irritable Bowel Syndrome: A Systematic Review and Meta-Analysis

**DOI:** 10.3390/jcm10163497

**Published:** 2021-08-08

**Authors:** Charlotte Le Morvan de Sequeira, Marie Kaeber, Sila Elif Cekin, Paul Enck, Isabelle Mack

**Affiliations:** Department of Psychosomatic Medicine and Psychotherapy, University Medical Hospital, 72076 Tübingen, Germany; charlotte.le-morvan-de-sequeira@student.uni-tuebingen.de (C.L.M.d.S.); marie.kaeber@student.uni-tuebingen.de (M.K.); sila.cekin@student.uni-tuebingen.de (S.E.C.); paul.enck@uni-tuebingen.de (P.E.)

**Keywords:** irritable bowel syndrome (IBS), functional gastrointestinal disorders (FGID), probiotics, paraprobiotics, bacterial lysate, quality of life (QoL), depression, anxiety, central nervous system (CNS), brain

## Abstract

Background: Functional gastrointestinal disorders such as irritable bowel syndrome (IBS) report clinical improvement following probiotic therapy, but whether psychiatric comorbidity and quality-of-life in IBS improves directly or in directly is unknown. This meta-analysis synthesized the evidence regarding the effects of probiotics on quality of life (QoL), anxiety and depression in IBS. Methods: The review was executed according to the Preferred Reporting Items for Systematic Reviews and Meta-Analyses guidelines using the databases PubMed, Web of Science and Cochrane Library. For QoL, the data were meta-analyzed, and for anxiety and depression a qualitative analysis was performed. Results: Thirty-five placebo-controlled studies were included of which 11 were eligible for meta-analysis. QoL improved with probiotic and placebo similarly, with the probiotic interventions slightly superior (mean QoL difference—0.36 (95% CI: 0.07, 0.64); *p* = 0.01). Anxiety and depression were similar between placebo and probiotic groups following therapy. Conclusion: For IBS, probiotic therapy improved QoL, but had no effects on anxiety and depression. However, the applied probiotics were not developed for selective effects on psyche and the brain. Therefore, it remains to be shown whether or not patients with IBS would benefit from second generation probiotics developed for these central effects (psychobiotics).

## 1. Introduction

Irritable bowel syndrome (IBS) belongs to the functional gastrointestinal disorders. The key symptoms are recurrent abdominal pain associated with defecation or a change in bowel habits [1]. The IBS subgroups are patients with either diarrhea predominance (IBS-D), with predominant constipation (IBS-C), with mixed or alternating bowel habits (IBS-M), or un-subtyped IBS (IBS-U) [1]. The pooled population prevalence is high—at 11.2% (95% CI: 9.8–12.8) [2]. IBS is often associated with other somatic comorbidities and psychiatric conditions such as anxiety and depression. A recent meta-analysis found the odds ratio for anxiety and depression to be three-fold when compared to healthy people [2]. These findings may explain why IBS is also associated with impaired quality of life, an increased use of the health care system and reduced work productivity [3,4].

IBS treatment focuses on improving the symptoms, since the underlying IBS etiology is not completely understood. Nevertheless, associations between IBS and alterations of the gastrointestinal microbiota, as well as increased incidences of IBS following acute gastrointestinal infections or use of antibiotics, are well described [5].

The GI microbiota is defined as the entirety of living microorganisms (bacteria, arachea and eucaryotes) that colonize the GI tract of a host organism [6]. Since exposure of the gut to numerous potential pathogens is common, it is imperative for the host to prevent their uncontrolled penetration into the body. Immune defense via unspecific strategies, along with gut-associated lymphoid tissue, are key factors in this interplay. Besides, many, mostly beneficial, interactions between the host and the indigenous microorganisms have been reported. The latter are important for maintaining the gut barrier function and overall health of the host [7,8]. Additionally, the gut microbiota impacts on central nervous system (CNS) function by modulating signaling pathways via the microbiota-gut-brain axis [9].

These are the reasons why therapeutics targeting the gastrointestinal microbiota, such as probiotics and paraprobiotics (inactivated bacteria or their fractions) [10,11], are of potential interest for treatment of IBS [1]. However, outcomes of therapy trials with viable and non-viable bacterial compounds in IBS are conflicting, for several reasons. One is that most probiotics are marketed as nutritional supplements [12] and not as drugs [10], and, in consequence, clinical trials usually do not match all standards imposed by the US Food and Drug Administration (FDA) or the European Medical Agency (EMA). Another critical issue is that not all probiotics may be of similar efficacy, and they may not be effective in all IBS subgroups to the same degree [10]. Whereas primary endpoints in IBS studies usually are FDA and EMA-defined symptom improvements [13,14], secondary outcomes frequently include quality-of-life (QoL) measures. However, the reporting of QoL outcomes is often lacks specificity, and it remains unclear whether QoL follows symptom improvement or not, or whether it represents an independent overall measure of the efficacy of the probiotic (and other) interventions on psychiatric and other CNS function measures.

Therefore, the present systematic review and meta-analysis aimed at providing a synthesis of the evidence regarding the effect of probiotics and paraprobiotics on QoL, psychiatric symptoms (anxiety and depression) and central functions—the latter defined as neurophysiological parameters measured, e.g., functional magnetic resonance imaging (fMRI)—in patients with IBS.

## 2. Materials and Methods

### 2.1. Literature Information Sources and Search Strategy

This review was developed and executed according to the Preferred Reporting Items for Systematic Reviews and Meta-Analyses (PRISMA) guidelines [15]. To identify all relevant studies examining the effect of probiotics on QoL and CNS function in patients, the databases PubMed, Web of Science and Cochrane Library were searched on the 20 April 2021. The protocol of this systematic review is registered on the PROSPERO platform with the registration number CRD42021253076. The full search strategy is documented in the Supporting Information Text S1, and consists of the three modules probiotics, IBS symptoms and QoL.

### 2.2. Eligibility Criteria

Eligibility criteria were based on the five PICOS dimensions, i.e., participants (P), interventions (I), comparators (C), outcome (O) and study design (S) [16].

Participants: Participants included adults of both sexes and of all ages with IBS.

Interventions: Eligible trials assessed the use of viable and non-viable microorganisms (single and multi-strain probiotics) or microbial cell extracts (bacterial lysates, sometimes called paraprobiotics), including second-generation probiotics developed for improving psychiatric conditions and potentially acting on CNS functions (also called psychobiotics), with a minimum of 3 weeks treatment. Studies applying prebiotics, synbiotics or antibiotics were excluded.

Comparators: Studies were eligible if a placebo control group was included.

Outcome Measures: Primary outcomes: QoL and psychiatric symptoms (anxiety and depression) measured with validated questionnaires and central function including changes in neurophysiological parameters measured by fMRI or electroencephalogram (EEG). Secondary outcomes: IBS symptoms according to ROME criteria [3,4].

Study design: Randomized, double-blind, placebo-controlled trials.

### 2.3. Study Selection, Data Collection and Organization

To identify eligible studies, the search results of the databases were combined and the duplicates were removed. Next, the titles and abstracts were screened. Full-text articles were evaluated regarding their eligibility (CLMdS and IM), with uncertainties being discussed between the authors (<3%). In the case of discrepancies, a third author was involved (PE).

The studies were classified into 2 groups:

- Group 1—Probiotics and QoL
- Group 2—Probiotics, anxiety and depression

### 2.4. Data Items and Statistics

The following information was extracted from each included article: year of publication, country of origin, study type, probiotic intervention, method for data collection, study outcomes including quality of life, sample characteristics (including sample size, sex, age), and sample size. Characteristics across studies are presented as frequency and per cent (%) or median [interquartile range], minimum and maximum for sample size, intake time, age and sex.

For QoL, the data were evaluated qualitatively and quantitatively (meta-analysis). The qualitative analyses allowed us to summarize all findings for their direction of change between the groups, because not all studies provided sufficient data and/or the applied measurements were heterogeneous. For the meta-analysis, a random-effect model was applied [17,18] using the software package Review Manager, version 5.4 [19] and QoL data of IBS-QoL (irritable bowel specific quality-of-life questionnaire [20]) are presented as mean and SD separately for the intervention and control groups, and the difference is expressed as mean difference and 95% confidence interval; it is displayed in forest plots.

Statistical heterogeneity was examined by visual inspection of forest plots and using the I^2^ statistics to quantify inconsistency between the studies. To reduce heterogeneity, subgroup analyses were performed for intake period (4–6 weeks versus 7–24 weeks intake time), efficacy for IBS symptom improvement and the type of probiotic preparation (single versus multi-strain probiotics).

Data on depression and anxiety were evaluated qualitatively because different assessment tools were used, and most studies did not report individual or group data but only stated that either differences or no differences were found between the groups.

Authors were contacted in case of missing data and 50% (2 out of 4) responded to the inquiry.

### 2.5. Risk of Bias

For all eligible studies, a risk of bias assessment was conducted using the Cochrane risk-of-bias tool for randomized trials (RoB 2) [21]. The tool consists of 5 domains addressing different types of bias: randomization process, deviations from the intended interventions, missing outcome data, measurement of the outcome and selection of the reported result. In each domain, appropriate questions must be answered for each single study. Next, the RoB2 algorithm is applied which evaluates the risks of the individual domains. Finally, an overall risk is calculated and expressed as “low” or “high” risk of bias, or the judgment can be expressed with “some concerns”.

## 3. Results

### 3.1. Study Selection and Categorization

**Figure 1:**
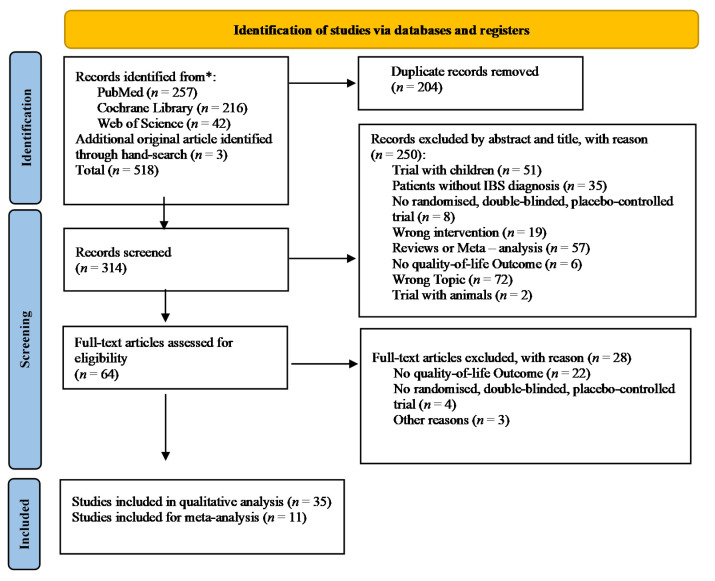
PRISMA flow chart for study inclusion.

The literature search process used to identify eligible studies is shown in Figure 1. Out of 518 identified studies, 35 studies remained for analysis.

### 3.2. Summary of Study Characteristics

An overview of the characteristics for the single trials is presented Table 1. The characteristics across the studies are given below.

The studies were published between 2005 and 2021. Most studies were conducted in Europe (*n* = 19; 54%) [22,23,24,25,26,27,28,29,30,31,32,33,34,35,36,37,38,39,40], followed by Asia (*n* = 11; 31%) [41,42,43,44,45,46,47,48,49,50,51], America (*n* = 4; 11%) [52,53,54,55] and Africa (*n* = 1; 3%) [56]. In total, the 35 trials included 4717 participants. The median age was 42 [41,42,43,44,45,46] years and 68% of the participants were women. The duration of the interventions ranged between 4 and 24 weeks with a median length of 7 [5.5–8] weeks. Most probiotics were applicated as capsules (*n* = 19; 54%) followed by liquids, e.g., milk (*n* = 6; 17%) and applications with sachets and pills (each *n* = 5; 14%). The daily intake of the probiotics ranged from one to four applications per day. Mostly, probiotic intake was once per day (*n* = 16; 46%) followed by twice a day (*n* = 13; 37%). There were single-strain (*n* = 20; 57%) and multi-strain probiotic studies (*n* = 15; 43%), the latter consisting of a range between two and eight different probiotic strains. The median number of colony-forming units (CFUs) was 1 × 10^10^ [4.4 × 10^9^ − 3.4 × 10^10^] CFUs per day with a range from 1 × 10^8^ to 9 × 10^11^ CFUs per day.

In most trials, QoL was a secondary endpoint, and only five studies (14%) considered QoL as primary endpoint. Many trials compared one probiotic group with the placebo group, but there were eight (23%) studies, which compared the results of the placebo group with results from several probiotic groups with different doses or species. The study with most probiotic groups had four different groups [37]. One study had a cross-over design whereas all other studies used parallel group designs [24].

Only one study investigated effects on CNS function by using functional magnetic resonance imaging. Few studies investigated anxiety and depression by validated questionnaires, such as the State and Trait Anxiety Inventory Questionnaire [53], the Hospital Anxiety and Depression Scale (n:8, 23%), the Hamilton Rating Scale for Depression and the Montgomery—Asberg Depression Rating Scale. Investigations were also performed with the Perceived Stress Scale (n:2; 6%) and the Dementia-Revised Memory and Behaviour Problem Checklist [46].

For the sample in the meta-analysis, 1977 participants were included in the 11 trials. The median age was 44 [42.5–45.5] years and 62% of the participants were women. The duration of the interventions ranged between 4 and 24 weeks with a median length of 6 [6,7,8,9,10] weeks. Most probiotics were applicated as capsules (*n* = 8; 73%) and consisted of single-strains (*n* = 5; 46%) and multi-strains (*n* = 6; 55%), the latter consisting of a range between two and seven different probiotic strains. The daily intake of the intervention ranged from one two four applications per day. Mostly, it was one application per day (*n* = 6; 55%) followed by twice a day (*n* = 3; 27%). The median number of colony-forming units (CFU) was 1.4 × 10^10^ [1 × 10^10^ − 3.5 × 10^10^] CFU per day with a range from 1.4 × 10^8^ to 4 × 10^11^ CFU per day.

### 3.3. Summary of Study Outcomes

Overall, the heterogeneity of studies was high with respect to probiotic species, application, intake period, inclusion criteria and outcomes.

**Figure 2:**
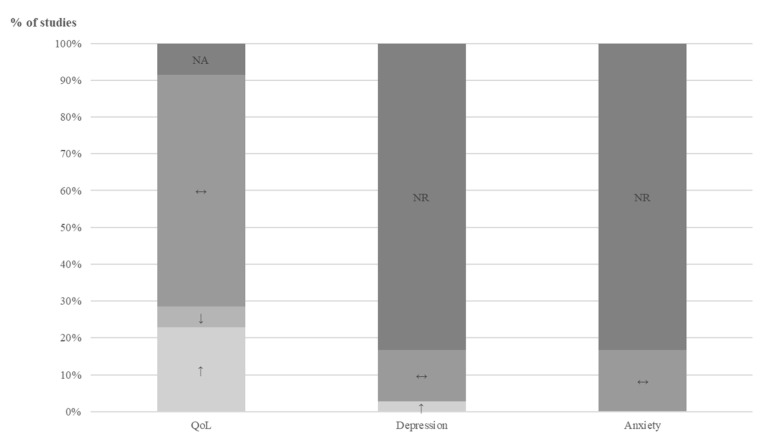
Changes of quality of life and symptoms for depression and anxiety compared between probiotic versus placebo intervention across studies. NR: not reported; NA: not applicable; QoL: quality of life; ↔: no significant differences between groups; ↑: improvement; ↓: deterioration.

*Quality of life:* Overall, quality of life improved in both groups, regardless of group allocation. For the group comparisons (probiotic versus placebo treatment) at qualitative level, all 35 studies were included and the results for the single studies are presented as overview in Table 2, in detail in Table S1 in Supplement Materials and across studies in Figure 2 Twenty-one studies (60%) showed no differences between the groups, nine studies were in favor of the probiotic group (26%), three studies reported only subgroup data (9%) and two studies only performed descriptive statistics (6%). Although the last five mentioned studies could not be included in the summary at qualitative level in Figure 6, they were included for completeness.

**Figure 3:**
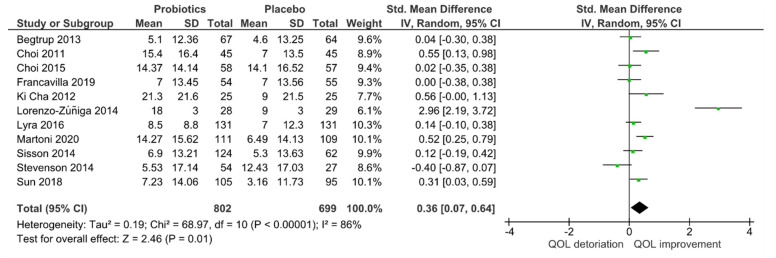
Quantitative analysis for QoL of randomized controlled trials in IBS receiving either probiotics or placebo treatment.

For quantitative analysis, 11 studies remained, and the results are presented as forest plots in Figure 2. The probiotic interventions were slightly favorable when compared to the placebo groups (mean QoL difference-0.36 (95% CI: 0.07, 0.64); *p* = 0.01). However, the heterogeneity was high with I^2^ = 86% in Figure 3, despite the applied random effect model. To reduce heterogeneity, subgroup analyses were performed.

**Figure 4:**
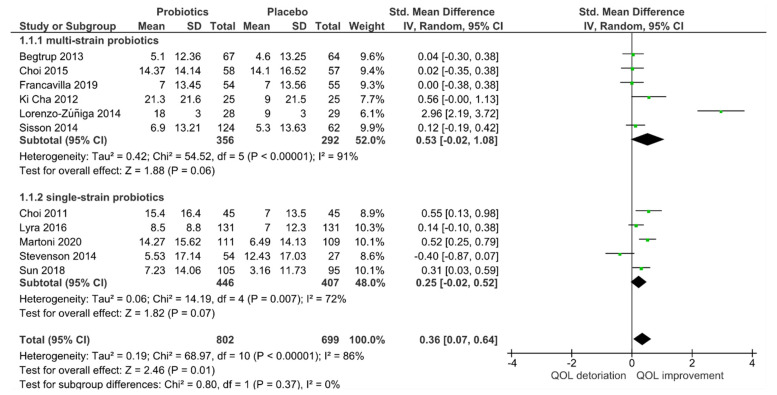
Effect of single-strain versus multi-strain probiotics on the quality of life (QoL) in randomized controlled trials for IBS receiving either probiotics or placebo treatment.

In the subgroup analysis of single-strain versus multi-strain studies in Figure 4, no significant subgroup effects were found (*p* = 0.37). Thus, the number of strains had no influence on the outcome of QoL. However, heterogeneity decreased slightly when analyzing only single-strain probiotic studies (I^2^ = 72%) but remained similarly high for multi-strain probiotic studies (I^2^ = 91%).

**Figure 5:**
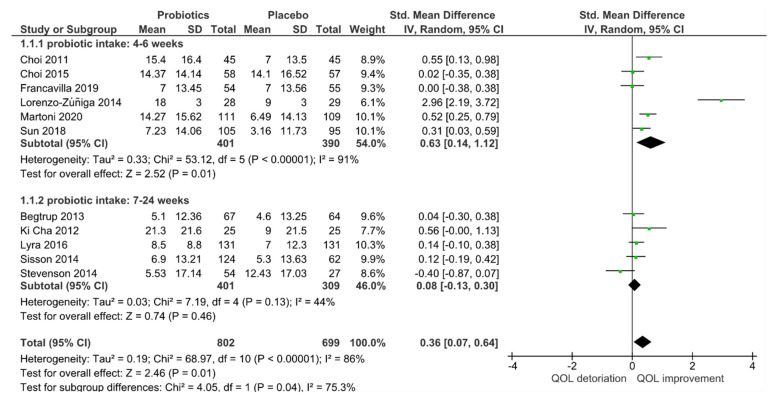
Effect of intake period on quality of life (QoL) of randomized controlled trials in IBS receiving either probiotics or placebo treatment.

In contrast, the intake period modified the effect of probiotic intake (*p* = 0.04) in Figure 5, with short-term applications favoring probiotics (*p* = 0.01) when compared to mid- to long-term applications. However, the heterogeneity for short-term intake of probiotics (I^2^ = 91%) remained high. In contrast, acceptable heterogeneity for mid to long-term applications (I^2^ = 44%) was achieved; at the same time no superiority for probiotics was evident anymore (*p* = 0.46).

**Figure 6:**
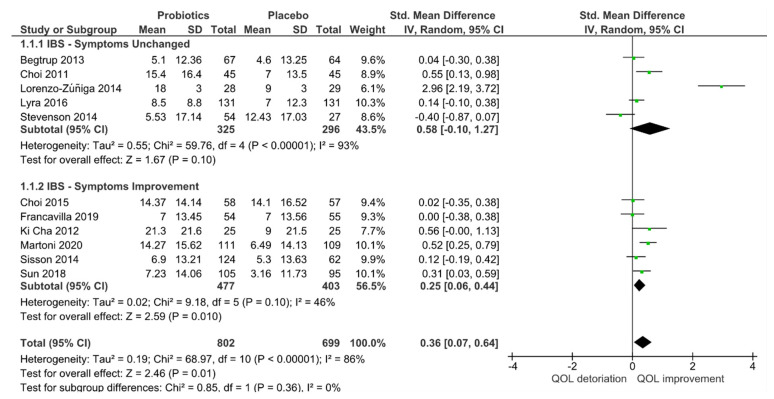
Effect of IBS symptom improvement on quality of life (QoL) of randomized controlled trials in IBS receiving either probiotics or placebo treatment.

Although no subgroup effects were observed when separating trials into “with” and “without” IBS symptom improvement (*p* = 0.36) groups in Figure 6, for studies with IBS improvement the heterogeneity was reduced (I^2^ = 46%), and QoL efficacy was still in favor of the probiotic group (*p* = 0.01). In contrast, trials with no IBS improvement remained high in heterogeneity (I^2^ = 93%), and no efficacy for QoL remained (*p* = 0.10).

*Anxiety, Depression and CNS Function*: Symptoms for anxiety and depression were summarized at the qualitative level since most studies reported no or insufficient data and only stated the direction of change or that no differences between the groups were found. The results are presented in Table 3 for each study separately and as summary across the studies in Figure 6. Detailed information is found in Supplement Materials 1 in Table S2. Overall, no symptom changes between intervention and placebo groups were reported. Only one study investigated CNS function using fMRI, in the probiotic *Bifidobacterium longum NCC3001* 6 week treatment group [53]. The fMRI analysis showed that BL reduced responses to negative emotional stimuli in multiple brain areas, including amygdala and fronto-limbic regions, compared with placebo.

### 3.4. Risk of Bias

**Figure 7:**
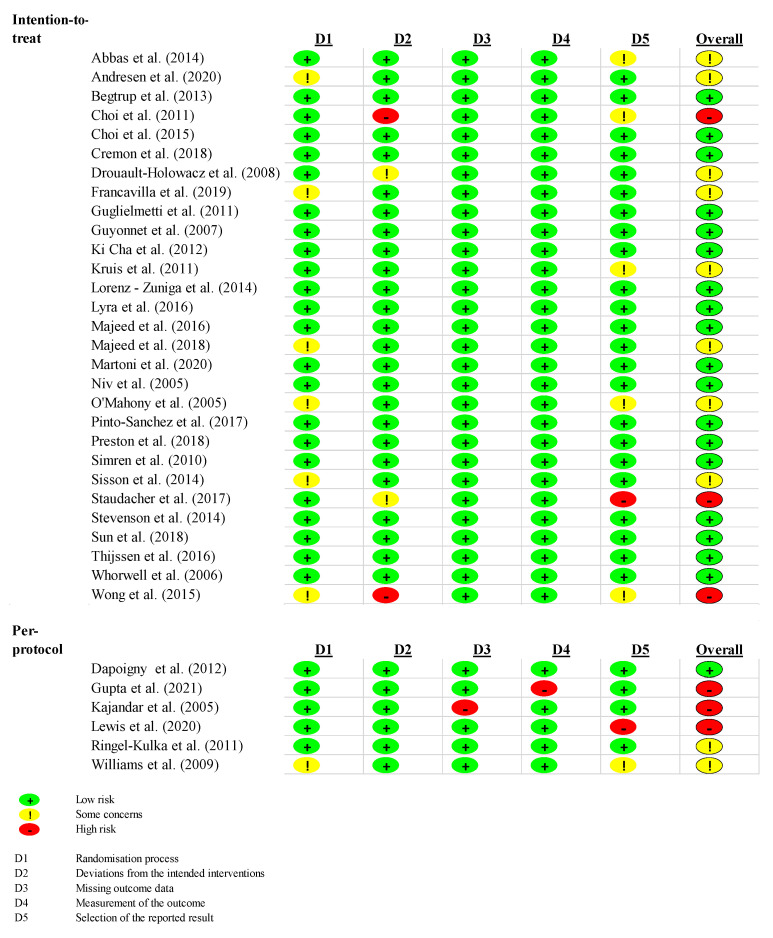
Risk of bias.

The risk of bias assessment is presented in Figure 7. The overall risk of bias was low in 19 studies (54%), with some concerns in 10 studies (29%) and high in six studies (17%). Concerning the studies in the meta-analysis, the overall risk of bias was low in eight studies (73%), with some concern in two studies (18%) and high in one study (9%).

Six of the trials were analyzed per protocol and not per intention-to-treat. There, the deviations from the intended intervention were all low, but the overall risk of bias was high in three studies (50%).

## 4. Discussion

In this systematic review and meta-analysis, the effects of probiotic treatment of patients with IBS on QoL and symptoms for depression and anxiety were investigated. At the qualitative level we found that probiotic treatment was not superior to placebo treatment with regards to QoL. Only a fraction of these studies was included in the meta-analysis following quality of study assessment. The results slightly favored the probiotic intervention, which is unlikely to have significant clinical relevance, especially when bearing in mind the high heterogeneity of studies. The heterogeneity improved especially for studies using single-strain probiotics, studies with intake periods longer than 6 weeks and those with significant improvement of IBS symptoms. Across all conditions, the efficacy of probiotics to improve QoL dropped. The reduced heterogeneity with single-strain probiotics may be due to the fact that the mechanism of action of a single strain may be more specific in contrast to multi-strain probiotics, where amplifying or weakening interactions between the different species may affect the outcome. However, interactions between probiotic species in traditional multi-strain preparations have rarely been characterized or investigated. The reduction in the heterogeneity of studies where IBS symptom relief was in favor of the probiotic group is most likely due to the somatic relief which is generally also accompanied by an improved QoL [20].

The meta-analysis by Zamani [2] reported that IBS patients have a higher risk for psychiatric comorbidities when compared to healthy participants, and some probiotics are known to have effects on CNS function in rodents and humans, communicating via the microbiota-gut-brain axis [9,57,58]. Therefore, we also investigated whether or not the applied probiotic species had potential psychiatric effects, limited to anxiety and depression. Overall, the results are in line with the findings for QoL. Symptoms of depression and anxiety did not differentiate between the groups after treatment in the qualitative analyses. One out of the 35 studies analyzed *Bifidobacterium longum NCC3001* for potential CNS effects using fMRI and found reduced limbic reactivity towards fearful stimuli. Thus, this could be a potential psychobiotic for IBS treatment, but adequately powered trials and further evidence is currently missing [59,60].

All names of the bacteria species used in each study are found in Table S3 in Supplement Materials 1 while all questionnaires applied by the included studies are shown in Table S4.

To date it is unclear whether or not patients with IBS may benefit from treatment with second generation probiotics selected for their CNS action [9]; aiming at improvement of the psychiatric outcome in particular, since the somatic IBS burden and psychological well-being are closely interrelated.

In addition, probiotics by themselves may not provide satisfying results in IBS symptom relief or QoL, warranting a multicomponent treatment of IBS, e.g., a combination of probiotics with diet and lifestyle changes. Only one study in this meta-analysis compared the effect of probiotics versus placebo with a low FODMAP diet versus sham diet in a 2 × 2 factorial design in 104 participants [37]. The authors found some beneficial improvements in IBS symptoms and QoL for the FODMAP diet, but not the probiotic condition. However, to answer this research question this study appeared to be underpowered, and to date the efficacy of such multicomponent treatments with regard to QoL, depression, anxiety and CNS function is unclear.

This study has strength and limitations. A clear strength is the methodological approach taken according to PRISMA criteria. To provide homogeneity of the trials, the search was limited to RCTs in adults only using probiotics and paraprobiotics, but neither synbiotics nor prebiotics. On the other hand, we found that, despite clear eligibility criteria, the heterogeneity of the studies was high at the descriptive and meta-analytical levels. To counter this problem, subgroup analyses were performed, which reduced heterogeneity to some degree. Another limiting factor is that QoL was mostly reported as a secondary outcome and with different instruments; often the results were not properly reported, requiring 24 out of 35 studies to be excluded from the quantitative analysis. To account for this problem, we summarized the findings at a qualitative and quantitative level, with the results pointing towards the same direction. The effects of probiotics on anxiety and depression were rarely investigated and, if so, symptoms were based on validated questionnaires but not appropriately reported. In addition, there may be differences in the effects of probiotics on quality of life between the different IBS subtypes. However, the use of mixed sub-type study populations in several studies and the application of different Rome criteria put this distinction out of reach for this meta-analysis.

## 5. Conclusions

Overall, we found that in IBS, probiotics are slightly favorable for the improvement of QoL when compared to placebo. No effects of probiotics were evident for the improvement of anxiety and depression in IBS patients. It remains unclear whether or not patients with IBS would benefit from second-generation probiotics (sometimes called psychobiotics), selected and developed for their ability to improve psychiatric conditions and, potentially, other CNS functions.

## Figures and Tables

**Table 1:** Summarized Trial Characteristics. IBS symptom improvement in probiotic versus placebo group: ↑↑↑ = *p* < 0.001, ↑↑= *p* < 0.01, ↑ = *p* < 0.05; Symptom deterioration in probiotic versus placebo group: ↓↓↓ = *p* < 0.001, ↓↓ = *p* < 0.01, ↓ = *p* < 0.05; ↔: no group differences; NR: not reported; A: analyzed sample size; PR: probiotic group; PL: placebo group; CFU: colony forming unit; SD: standard deviation; f: female; IRN: Iran; NLD: Netherlands; ITA: Italy; DNK: Denmark; KOR: Korea; JPN: Japan; IRL: Ireland; FRA: France; SWE: Sweden; CAN: Canada; PAK: Pakistan; DEU: Deutschland; IND: India; SGP: Singapore; ISR: Israel; ESP: Spain; FIN: Finland; ZAF: South Africa; CHN: China.

Author (Year)	Country	Intake Length (Week)	Sample Size (A); Sex (f %); Age Mean (SD); Health Condition; Groups (N)	Probiotic Species (N); Dose; Frequency of Intake per Day; Application	Outcomes
Abbas, Z., et al. (2014)	PAK	6	72(72); f: 26.4%; age: PR -> 37.7 ± 11.6, PL -> 33.0 ± 12.0; Rome III for IBS-D; groups: PR (37), PL (35)	Saccharomyces boulardii (1); dose: NR; 1/day; liquid	IBS Symptoms: ↔ IBS-QoL: ↑↑ overall; ↑ body image + food avoidance
Andresen, V. et al. (2020)	DEU	8	443(443); f: 69.3%; age: PR -> 40.1 (12.8), PL -> 42.6 (13.8); Rome III; groups: PR (221), PL (222)	Bifidobacterium bifidum HI-MIMBb75 (1); 0.5 × 10^9^ CFU; 2/day; capsules	IBS -Symptoms: ↑↑↑composite response; ↑↑↑ AR; ↓ SGA; ↓↓ IBS-SSS; ↓ Abdominal pain; ↓ Distension or bloating + Pain associated with bowel movement; ↓↓ Discomfort SF-12: ↑
Begtrup, L. M., et al. (2013)	DNK	6 months	131(131); f: 74%; age: 30.52 (9.42); Rome III; groups: PR (67), PL (64)	provided by Arla Foods (3); 1.3 × 10^10^ CFU; 4 /day; capsules	GSRS-IBS: 3 months ↔; 6 months ↔ IBS—QoL: ↔
Choi, C. H., et al. (2011)	KOR	4	90(NR); f: 51.1%; age: 41 ± 13; Rome II; groups: PR (45), PL (45)	Saccharomyces boulardii (1); 2 × 10^11^ CFU; 2/day; capsules	IBS symptoms: ↔ IBS-QoL: ↑ overall; domain: ↑↑ interference with activity, ↑ social reaction
Choi, C. H., et al. (2015)	KOR	6	286(286); f: 50.5%; age: 47; Rome III; groups: PR1 (54), PR2 (60), PR3 (58), PR4 (56), PL (57)	Medilac (2); PR1: 1 × 10^10^ CFU/10 mg, PR2: 1.5 × 10^10^ CFU/10 mg, PR3: 1.5 × 10^10^ CFU/15 mg, PR4: 3 × 10^10^ CFU/15 mg; 1/day; pills	AR: ↑ week 3 + 4 SAG: ↔ Likert Scales IBS symptoms: ↔ IBS-QoL: ↔
Cremon, C. et al. (2018)	ITA	4	42(40); f: 65%; age: PR-PL -> 37.35 ± 11.25, PL-PR -> 44.55 ± 12.98; Rome III; groups: PR-PL (22), PL-PR (20); RCT Cross over	Lactobacillus paracasei CNCM I1572 (1); 2.4 × 10^10^ CFU; 2/day; capsules	Abdominal pain/discomfort, IBS degree of relief: ↔VAS satisfaction with treatment: ↔ HADS: ↔ SF-12: ↔
Dapoigny, M. et al. (2012)	FRA	4	52(50); f: 70%; age: PR -> 46.1 ± 11.3, PL -> 48.0 ± 10.8; Rome III; groups: PR (25), PL (25)	Lactobacillus casei variety rhamnosus (1); 2 × 10^8^ CFU; 3/day; capsules	IBS-SSS: ↔GIQLI: ↔HAD: ↔
Drouault-Holowacz, S., et al. (2008)	FRA	4	106(100); f: 76%; age:46; Rome II; groups: PR (48), PL (52)	sponsored by PiLeJe (4); 1 × 10^10^ CFU; 1/day; powder	overall IBS symptoms: abdominal pain score: ↓ between week 1 + 4 (↓ A-IBS group) IBS specific FDD-quality-of-life: ↔ SF-36: ↔
Francavilla, R. et al. (2019)	ITA	6	109(NR); f: NR%; age: PR -> 43.3 (18.8–62.2), PL -> 44.6 (19.3–63.4); ROME III criteria and long term treated CD (Celiac Disease) with GFD (gluten-free diet); groups: PR (54), PL (55)	provided by Probioresearch (5); 4 × 10^10^ CFU; 1/day; sachet	IBS-SSS: ↓↓↓GSRS: ↓↓↓ IBS QoL: ↔
Guglielmetti, S., et al. (2011)	DEU	4	122(122); f: 67.2%; age: PR -> 36.65 ± 12.42, PL -> 40.98 ± 12.80; Rome III; groups: PR (60), PL (62)	Bifidobacterium bifidum MIMBb75 (1); 1 × 10^9^ CFU; 1/day; capsules	SGA: ↓↓↓IBS Symptoms: ↓↓↓ pain/discomfort, distension/bloating, urgency, bowel movement satisfactionoverall responders: ↓↓↓ AR: ↓↓↓SF-12: ↑ physical health, ↑↑ mental health
Gupta, A. K. et al. (2021)	IND	80 days	40(38); f: 30%; age: PR -> 36.20 ± 9.81, PL -> 34.80 ± 11.06; Rome IV; groups: PR (20), PL (20)	Bacillus coagulans LBSC (1); 2 × 10^9^ CFU/g/sachet; 3/day; sachet	DSFQ: ↓ Bloating and cramping, Stomach rumbling, Vomiting, Anxiety; ↓↓↓ Abdominal pain, Headache; ↓↓ Diarrhoea and constipation, Nausea IBS-SSS/QoL-Questionnaire: no comparison between two groups with significance calculation
Guyonnet, D., et al. (2007)	FRA	6	274(267); f: 74.5%; age: PR -> 49.4 ± 11.4, PL -> 49.2 ± 11.4; Rome II; groups: PR (135), PL (132)	provided by Danone Research (3); 1.49 × 10^10^ CFU; 2/day; fermented milk	IBS Symptoms: ↔; ↓ bloating at week 3 HrQoL/FDDQL: ↔; ↑↑responders for the discomfort dimension at week 3
Kajander, K. et al. (2005)	FIN	6 months	103(81); f: 76.7%; age: PR -> 46, PL -> 45; Rome I, majority Rome II; groups: PR (52), PL (51)	from Valio Ltd. (3); 8–9 × 10^9^ CFU; 1/day; capsules	Intensity of GI symptoms in month 4–6: ↓ total, ↓↓ borborygmi, ↓ urgency, ↓ incomplete evacuation SF-36-QoL:↔
Ki Cha, B., et al. (2012)	KOR	8	50(50); f: 48%; age: 39.7; Rome III criteria, included D-IBS, excluded C-IBS or mixed-types; groups: PR (25), PL (25)	Duolac7 (7); 1.0 × 10^10^ cells; 2/day; capsules	AR: ↑AR (↑↑ AR responders for ≥5 weeks) VAS Score for IBS Symptoms: ↔ IBS-QoL: ↔, ↑ Health worry score
Kruis, W. et al. (2012)	DEU	12	120(120); f: 76.7%; age: PR -> 46.3 ± 12.1, PL -> 45.1 ± 12.7; Rome II + ≥ 26 points Kruis score; groups: PR (60), PL (60)	MUTAFLOR (1); 2.5–25 × 10^9^ CFU; first 4 days: 1/day, after 4 days:2/day; capsules	Rate of clinical response: ↑↑ week 10, ↑ week 11 subgroup analyses: ↑↑ patients with prior bacterial intestinal infection (*n* = 5) ↑↑ patients with an altered enteric microflora IMPSS: ↔ HRQL: ↔
Lewis, E. et al. (2020)	CAN	8	285(251); f: 77.7%; age: PR-L.paracasei -> 42.42 ± 12.30 (84), PR-B. longum -> 42.31 ± 16.88 (86),PL -> 41.84 ± 16.14 (81); Rome III; groups: PR-L.paracasei (95), PR-B. longum (95),PL (95)	Bifidobacterium longum R0175 or Lactobacillus paracasei HA-196 (1); each 10 × 10^9^; 1/day; capsules	IBS-SSS: ↔ (PP) SF-36: ↔ IBS-QoL: NR HADS: ↔
Lorenzo-Zúñiga, V., et al. (2014)	ESP	6	84(71 -> QoL, VSI; 73 -> relief); f: 63.2%; age: PR-high dose -> 47.5 ± 13.1, PR-low dose -> 46.3 ± 11.6, PL -> 46.5 ± 13.1; Rome III with diarrhoea; groups: PR-high dose (28), PR-low dose (27), PL (29)	produced by ABbiotics (3); high dose: 1–3 × 10^10^ CFU, low dose: 3–6 × 10^9^ CFU; 1/day; capsules	VSI scale: ↑ both probiotic groups; ↔ between probiotic groupsSymptom relief: ↔ IBS-QoL: ↑ high dose probiotics after 3 weeks, ↑ high and low dose probiotics after 6 weeks domain: ↑ Mental Health both probiotic doses ↔ between doses
Lyra, A., et al. (2016)	FIN	12	391(391); f: 74.7%; age: PR-high dose -> 47.2 ± 12.5, PR-low dose -> 47.1 ± 13.3, PL -> 49.4 ± 12.9; Rome III; groups: PR-high dose (131), PR-low dose (129), PL (131)	Lactobacillus acidophilus NCFM (1); high dose: 1 × 10^10^ CFU, low dose: 1 ± 10^9^ CFU; 1/day; capsules	IBS-SSS: ↔, ↓ Subgroup moderate to severe pain (post-hoc) AR: ↔ IBS-QoL: ↔ HADS: ↔
Majeed, M., et al. (2016)	IND	90 days	36(30); f: 52.8%; age: PR -> 36.2 ± 11.07, PL -> 35.4 ± 10.75; Rome III for functional IBS; groups: PR (18), PL (18)	Bacillus coagulans MTCC 5856 (1); 2 × 10^9^ spores; 1/day; pills	GI-discomfort-Questionnaire: ↓↓ bloating, vomiting, diarrhoea + stool frequency, ↓↓↓ abdominal pain VAS score for abdominal pain: ↑↑ Physician’s global assessment score for disease severity: ↑↑IBS-QoL: ↑↑
Majeed, M., et al. (2018)	IND	90 days	40(40); f: 85%; age: PR -> 40.36 ± 10.28, PL -> 43.88 ± 9.85; Rome III for functional IBS; groups: PR (20), PL (20)	Bacillus coagulans MTCC 5856 (1); 2 × 10^9^ spores; 1/day; pills	GI-DQ: ↔ CGI-S: ↔ IBS-QoL: ↓ HAM-D: ↓ MADRS: ↓ CES-D: ↔ CGI-I: ↔ RMBPC: ↓Dementia total frequency scoring
Martoni, C. J., et al. (2020)	IND	6	336(330); f: 49.4%; age: PR-L. acidophilus -> 39.41 (11.80), PR-B. lactis -> 41.60 (11.11), PL -> 37.61 (10.12); Rome IV; groups: PR-L. acidophilus (111), PR-B. lactis (110), PL (109)	Lactobacillus acidophilus DDS^®^-1 or Bifidobacterium animalis subsp. lactis UABla-12™ (1); 1 × 10^10^ CFU; 1/day; capsules	IBS-SSS total: ↓↓↓ both probiotic groups, ↓ L. acidophilus group vs. B. lactis group APS-NRS: ↓↓ both probiotic groups, ↓↓↓ L. acidophilus group vs. B. lactis group IBS-SSS-QoL: ↑↑↑ L.acidophilus, ↑↑ B.lactis IBS-QoL: ↓L.acidophilus group PSS: ↓ L. acidophilus group
Niv, E., et al. (2005)	ISR	6 months	54(54); f: 66.7%; age: PR -> 45.7 + 14.2, PL -> 45.6 + 16.1; Rome II; groups: PR (27), PL (27)	Lactobacillus reuteri ATCC 5573 (1); 1 × 10^8^ CFU; first 7 days: 4/day, after day 7: 2/day; pills	IBS-SSS: ↔ IBS-QoL: ↔
O’Mahony, L., et al. (2005)	IRL	8	75(75); f: 64%; age: 44.3; Rome II; groups: PR-L.salivarius (NR), PR-B.infantis (NR), PL (NR)	Man/Rogosa/Sharp broth (1); 1 × 10^10^ CFU; 1/day; malted milk	IBS Symptoms: abdominal pain: ↓ B. infantis vs. placebo post-hoc: Composite: ↓ B. infantis vs. L. salivarius vs. placebo bowel movement difficulty: ↓ B. infantis vs. L. salivarius vs. placebo IBS-QoL: ↓ health worry for B. infantis
Pinto-Sanchez, M. I., et al. (2017)	CAN	6	44(ITT = 44/PP = 38); f: 54.5%; age (IQR): PR -> 46.5(30-58), PL -> 40.0 (26-57); Rome II + mild to moderate anxiety and/or depression; groups: PR (22), PL (22)	Bifidobacterium longum NCC3001 (1); 1.0 × 10^10^ CFU; 1/day; sachet	AR on IBS Symptoms: week 6 (PP)Brimingham IBS score total: ↔ SF-36: ↑ in physical subdomain (physical, physical function,role physical) HADS: ↔ STAI: ↔ fMRI: ↓ engagement of the amygdala and frontal + temporal cortices + ↑ engagement of occipital regions in response to fear stimuli
Preston, K., et al. (2018)	USA	12	113(113); f: 60.2%; age: PR -> 40.6 ± 13.4, PL -> 39.9 ± 14.0; Rome III; groups: PR (76), PL (37)	provided by Bio-K Plus International Inc. (3); 50 × 10^9^ CFU; 2/day; capsules	IBS-SSS, AR of IBS symptoms, IBS-QoL-Questionnaire: improvement in %, no significance calculation
Ringel-Kulka, T., et al. (2011)	USA	8	60(53); f: 72%; age: 37; Rome III; groups: PR (31), PL (29)	supported by K23 DK075621, RR00046 and Danisco USA Inc. (2); 1 × 10^11^ CFU; 2/day; pills	Global relief of GI: ↔Satisfaction with treatment: ↔ functional GI symptoms: ↓↓ bloating symptom (week 4) IBS-SSS: ↓ bloating severity scores (week 4) other endpoints (+ global well being): HR-QoL/IBS-QoL: ↔
Simrén, M., et al. (2010)	SWE	8	74(74); f: 70.3%; age: 42 ± 16; Rome II; groups: PR (37), PL (37)	supported by Arla Foods Innovation and the Faculty of Medicine, University of Göteborg (3); 5 × 10^7^ CFU ⁄ mL; 2 × 200 mL/day; fermented milk	IBS-SSS total: ↔ week 1) AR: ↔ GI symptom questionnaire: ↔ IBS-QoL: ↔ HAD: ↔
Sisson, G., et al. (2014)	UK	12	186(186); f: 69.4%; age: PR -> 39.1 (10.5), PL -> 36.8 (10.8); Rome III + at study begin symptomatic; groups: PR (124), PL (62)	Symprove (4); 1 × 10^10^ CFU; 1 mL/kg per day; suspension	IBS-SSS: ↓↓ total, ↓ subscore pain, ↓↓ subscore bowel habit IBS QoL: ↔
Staudacher, H. et al. (2017)	UK	4	104(104); f: 67.3%; age: Sham diet + PL -> 33 (12), Sham diet + PR -> 35 (11), low FODMAP diet + PL -> 36 (11), low FODMAP diet + PR -> 38 (13); Rome III; groups: Sham diet + PL(27), Sham diet + PR(26), low FODMAP diet + PL(24), low FODMAP diet + PR(27)	Vivomixx (Europe), Visbiome (USA) (8); 4.5 × 10^11^ bacteria; 2/day; sachet	GSRS: ↓ Flatulence IBS-SSS: ↔ AR: ↑ at follow-up SF-36: ↔ IBS-QoL: ↔
Stevenson, C., et al. (2014)	ZAF	8	81(81); f: 97.5%; age: PR -> 48.15 ± 13.48, PL -> 47.27 ± 12.15; Rome II no IBS-M ; groups: PR-D-IBS (27), Pr-C-IBS (27), PL (27)	L. plantarum 299 v (1); 5 × 10^9^ CFU; 2/day; capsules	Francis Severity Score (pain and distension): ↔ IBS-QoL: ↔
Sun, Y. Y., et al. (2018)	CHN	4	200(200) -> responder rate, 166 -> PP in symptom analysis); f: 84%; age: PR -> 43.00 (12.45), PL -> 44.91 (13.01); IBS-D by Rome III; groups: PR (105), PL (95)	Clostridium butyricum (1); 1.5 × 10^7^ CFU/g; 3 × 3/day; capsules	IBS-SSS: ↓ total; individual components: ↓ bowel habit, ↓ QoL satisfaction responder rate: ↑ (↑↑ moderate and sever symptoms) IBS-QoL: ↑ overall; individual components: ↑↑ activity interference, ↑↑↑ health worry
Thijssen, A. Y., et al. (2016)	NLD	8	80(80); f: 68.8%; age: PR -> 41.1 ± 14.8, PL -> 42.4 ± 13.5; Rome II; groups: PR (39), PL (41)	Lactobacillus casei Shirota (1); 6.5 × 10^9^ CFU; 2/day; fermented milk	MSS: ↔ after treatment; 8 week after treatment: ↓ for discomfort, flatulence and total responders: ↔QoL Physical composite score: ↔QoL mental composite score: ↔CSFBD: ↔
Whorwell, P. J., et al. (2006)	UK	4	362(362); f: 100%; age: PR-BIFIDO10 -> 41.8 (1.10), PR-BIFIDO8 -> 42.7 (1.10), PR-BIFIDO6 -> 40.8 (1.10), PL -> 42.4 (1.09); Rome II; groups: PR-BIFIDO10 (90), PR-BIFIDO8 (90), PR-BIFIDO6 (90), PL (92)	Bifidobacterium infantis (1); PR-BIFIDO10:1 × 10^10^, PR-BIFIDO8: 1 × 10^8^, PR-BIFIDO6: 1 × 10^6^; 1/day; capsules	Primary symptoms of IBS: ↓ probiotic group-BIFIDO8 in: Abdominal pain/discomfort, bloating/distention, Incomplete evacuation, Straining, Passage of gas, Bowel habit satisfaction, Composite score; ↓↓ probiotic group-BIFIDO8: Overall assessment of IBS symptoms SGA of relief for abdominal pain/discomfort + IBS symptoms: ↓ probiotic group-BIFIDO8 in IBS symptoms IBS-QoL: ↔HAD: ↔
Williams, E. A., et al. (2009)	UK	8	56(52); f: 86.5%; age: PR -> 40 (12), PL -> 38 (11); Rome II; groups: PR (28), PL (24)	prepared by Cultech Ltd. (4); 2.5 × 10^10^ CFU; 1/day; capsules	IBS-SSS: overall↓ week 6 + 8; Satisfaction with bowel habit ↓ week 6; Number of days with pain ↓ week 10 (follow up) IBS-SSS-QoL: ↓↓ week 8
Wong, R. K., et al. (2015)	SGP	6	42(42); f: 45.2%; age: PR -> 53.35 (4.15), PL -> 40.86 (3.51); Rome III; groups: PR (20), PL (22)	VSL#3 (8); 112.5 × 10^9^ CFU; 4 × 2/day; capsules	IBS-SSS: (↓ total --> male participants) ↓ abdominal pain duration score, ↓ abdominal distension severity scores SBDQ: NR Bowel Symptom Diary: NR IBS-SSS-QoL: ↔HAD: ↔ PSS: ↔

**Table 2:** QoL changes compared between probiotic versus placebo intervention. IBS: irritable bowel syndrome; QoL: quality of life; ↑↑: QoL improvement in probiotic versus placebo group: ↑↑= *p* < 0.01, ↑ = *p* < 0.05; QoL deterioration in probiotic versus placebo group: ↓ = *p* < 0.05; ↔: no group differences and no trend in QoL; ↔ (+): no significant group differences (trend towards QoL improvement); ↔ (−): no significant differences between groups (trend QoL towards deterioration); NA: not applicable.

	Year	Questionnaire	Result of Subgroups	Total Result
Abbas, Z., et al.	2014	IBS - QOL	-	↑↑
Andresen, V. et al.	2020	SF - 12 sum score	-	↑
Begtrup, L. M., et al.	2013	IBS - QOL	-	↔ (+)
Choi, C. H., et al.	2011	IBS - QOL	-	↑
Choi, C. H., et al.	2015	IBS - QOL	-	↔ (+)
Cremon, C. et al.	2018	SF-12	-	↔
Dapoigny, M. et al.	2012	GIQLI	-	↔
Drouault-Holowacz, S., et al.	2008	IBS specific FDD-quality-of-life SF-36	-	↔ (+)
Francavilla, R. et al.	2019	IBS QOL	-	↔ (-)
Guglielmetti, S., et al.	2011	SF-12	↑↑ (mental), ↑ (physical)	NA
Gupta, A. K. et al.	2021	QoL - Questionnaire	-	NA
Guyonnet, D., et al.	2007	FDDQL - discomfort	-	↔ (+)
Kajander, K. et al.	2005	SF-36	-	↔ (NA)
Ki Cha, B., et al.	2012	IBS-QOL	-	↔ (+)
Kruis, W. et al.	2012	HRQL	-	↔ (+)
Lewis, E. et al.	2020	SF - 36 IBS - QOL	-	↔ (+)
Lorenzo-Zúñiga, V., et al.	2014	IBS - QOL	-	↑
Lyra, A., et al.	2016	IBS-QOL	-	↔ (+)
Majeed, M., et al.	2016	IBS-QOL	-	↑↑
Majeed, M., et al.	2018	IBS-QOL	-	↓
Martoni, C. J., et al.	2020	IBS-QOL	-	↓
Niv, E., et al.	2005	IBS-QOL	-	↔ (-)
O’Mahony, L., et al.	2005	IBS-QOL	↓ in "health worry" for B. infantis	NA
Pinto-Sanchez, M. I., et al.	2017	SF-36	↑ in physical subdomain	NA
Preston, K., et al.	2018	IBS-QOL-Questionnaire	-	NA
Ringel-Kulka, T., et al.	2011	IBS-QOL	-	↔ (+)
Simrén, M., et al.	2010	IBS-QOL	-	↔ (+)
Sisson, G., et al.	2014	IBS-QOL	-	↔ (+)
Staudacher, H. et al.	2017	IBS-QOL SF-36	-	↔ (+)
Stevenson, C., et al.	2014	IBS-QOL	-	↔ (-)
Sun, Y. Y., et al.	2018	IBS-QOL	-	↑
Thijssen, A. Y., et al.	2016	QOL Physical composite scoreQOL mental composite score	-	↔
Whorwell, P. J., et al.	2006	IBS-QOL	-	↔ (NA)
Williams, E. A., et al.	2009	IBS-SSS-QOL	-	↑↑
Wong, R. K., et al.	2015	IBS-SSS-QOL	-	↔

**Table 3:** Changes of symptoms for depression and anxiety compared between probiotic versus placebo intervention; HADS = Hospital Anxiety and Depression Scale. ↑: symptom improvement *p* < 0.05 in probiotic versus placebo group; ↓: symptom deterioration *p* < 0.05 in probiotic versus placebo group; ↔: no group differences and no trend; ↔ (+): no group differences (trend towards symptom improvement); NR: not reported.

	Year	HADS Depression Score	HADS Anxiety Score	Total Score
Cremon, C. et al.	2018	↔ (+)	↔ (+)	NR
Dapoigny, M. et al.	2012	NR	NR	↔
Lewis, E. et al.	2020	NR	NR	↔
Lyra, A., et al.	2016	↔	↔	NR
Pinto-Sanchez, M. I., et al.	2017	↑	↔ (+)	NR
Simrén, M., et al.	2010	↔	↔	NR
Whorwell, P. J., et al.	2006	↔	↔	NR
Wong, R. K., et al.	2015	↔ (+)	↔ (+)	NR

## Data Availability

All data are reported in the manuscript and the supplementary material.

## Supplementary Materials

The following are available online at https://www.mdpi.com/article/10.3390/jcm10163497/s1, Text S1: Search Strategy, Table S1: Detailed Data of the Quality-of-Life Questionnaire, Table S2 Detailed Data of the Depression and Anxiety Score HADS, Table S3 Bacterial Species, Table S4: Questionnaires applied by the included studies.
