# Supplementary material for: The Effect of Probiotics on Quality of Life, Depression and Anxiety in Patients with Irritable Bowel Syndrome: A Systematic Review and Meta-Analysis
Source: J Clin Med. 2021 Aug 8;10(16):3497. doi: 10.3390/jcm10163497 (PMC8397103; doi:10.3390/jcm10163497)
Supplement: Supplementary file 1 [file jcm-10-03497-s001.zip › jcm-1329966-supplementary.pdf]

Effect of Probiotics on Quality of Life and Central Nervous System Functions in patients with Irritable Bowel Syndrome: A Systematic Review and Meta-Analysis

Charlotte Le Morvan de Sequeira, Marie Kaebler, Sila Elif Cekin, Paul Enck and Isabelle Mack

SUPPORTING INFORMATION:

Text S1: Search Strategy

PubMed-
(((("Healthy Volunteers"[Mesh] OR Healthy*[tiab]) OR ("Irritable Bowel Syndrome"[Mesh] OR irritable bowl Syndrome[tiab] OR irritable bowel Syndrome[tiab] OR IBS[tiab])) AND (("Probiotics"[Mesh] OR Probiotic*[tiab] OR Paraprobiotic*[tiab] OR bacterial lysate*[tiab] OR Psychobiotic*[tiab]) NOT (Synbiotic*[tiab] NOT ("Probiotics"[Mesh] OR Probiotic*[tiab] OR Paraprobiotic*[tiab] OR bacterial lysate*[tiab] OR Psychobiotic*[tiab])))) AND ("Treatment Outcome"[Mesh] OR Treatment Outcome[tiab] OR Clinical Effectiveness[tiab] OR Treatment Effectiveness[tiab] OR Treatment Efficacy[tiab] OR Clinical Efficacy[tiab] OR "Electroencephalography"[Mesh] OR EEG[tiab] OR Electroencephalogram*[tiab] OR Electroencephalograph*[tiab] OR "Magnetoencephalography"[Mesh] OR Magnetoencephalograph*[tiab] OR "Magnetic Resonance Imaging"[Mesh] OR Magnetic Resonance Imaging[tiab] OR NMR Imaging[tiab] OR NMR Tomography[tiab] OR MRI[tiab] OR Zeugmatography[tiab] OR Shift Imaging[tiab] OR “Functional MRI”[tiab] OR “Functional Magnetic Resonance Imaging”[tiab] OR ((Measur*[tiab] OR Test*[tiab]) AND (Behaviour[tiab] OR Behavior[tiab] OR Neuropsychologic*[tiab] OR Psychometric*[tiab])))) AND (("Randomized Controlled Trial" [Publication Type] OR (randomized[tiab] AND controlled[tiab] AND trial*[tiab]) OR (Placebo[tiab] AND controlled[tiab] AND trial*[tiab])) OR ("Double-Blind Method"[Mesh] OR Double Blind Method[tiab] OR Double Blind Methods[tiab] OR Double Masked Method[tiab] OR Double Masked Methods[tiab] OR Double Masked Stud*[tiab] OR Double Blind Stud*[tiab]))

Cochrane Library-
[mh "Healthy Volunteers"] OR (Healthy*):ti,ab,kw [mh "Irritable Bowel Syndrome"] OR ("irritable bow?l Syndrome" OR IBS):ti,ab,kw ([mh "Probiotics"] OR (Probiotic* OR Paraprobiotic* OR "bacterial lysate*" OR Psychobiotic*):ti,ab,kw) NOT ((Synbiotic*):ti,ab,kw NOT ([mh "Probiotics"] OR (Probiotic* OR Paraprobiotic* OR "bacterial lysate*" OR Psychobiotic*):ti,ab,kw)) [mh "Treatment Outcome"] OR ("Treatment Outcome" OR "Clinical Effectiveness" OR "Treatment Effectiveness" OR "Treatment Efficacy" OR "Clinical Efficacy"):ti,ab,kw OR [mh "Electroencephalography"] OR (EEG OR Electroencephalogram* OR Electroencephalograph*):ti,ab,kw OR [mh "Magnetoencephalography"] OR (Magnetoencephalograph*):ti,ab,kw OR [mh "Magnetic Resonance Imaging"] OR ("Magnetic Resonance Imaging" OR"NMR Imaging" OR "NMR Tomography" OR MRI OR Zeugmatography OR "Shift Imaging" OR "Functional MRI" OR "Functional Magnetic Resonance Imaging"):ti,ab,kw OR (((Measur* OR Test*):ti,ab,kw) AND ((Behavio?r OR Neuropsychologic* OR Psychometric*):ti,ab,kw)) (Randomized Controlled Trial):pt OR ((randomized NEAR/0 controlled NEAR/0 trial*):ti,ab,kw) OR ((Placebo NEAR/0 controlled NEAR/0 trial*):ti,ab,kw) OR [mh "Double-Blind Method"] OR ("Double Blind Method*" OR "Double Masked Method*" OR "Double Masked Stud*" OR "Double Blind Stud*"):ti,ab,kw

Web of Science-
TS=(Healthy NEAR/3 Volunteer*) TS=("Irritable Bow?l Syndrome" OR IBS) TS=((Probiotic* OR Paraprobiotic* OR “bacterial lysate*” OR Psychobiotic*) NOT (Synbiotic* NOT (Probiotic* OR Paraprobiotic* OR “bacterial lysate*” OR Psychobiotic*))) TS=("Treatment Outcome" OR ((Clinical OR Treatment) NEAR/2 (Effectiveness OR Efficacy)) OR Electroencephalography OR EEG OR Electroencephalogram* OR Electroencephalograph* OR Magnetoencephalograph* OR “Magnetic Resonance Imaging” OR “NMR Imaging” OR “NMR Tomography” OR MRI OR Zeugmatography OR “Shift Imaging*” OR “Functional Magnetic Resonance Imaging” OR ((Measur* OR Test*) NEAR/5 (Behavio?r OR Neuropsychologic* OR Psychometric*))) TS=("Randomized Controlled Trial" OR (randomized NEAR/2 controlled NEAR/2 trial*) OR (Placebo NEAR/2 controlled NEAR/2 trial*) OR “Double Blind Method*” OR “Double Masked Method*” OR “Double Masked Stud*” OR “Double Blind Stud*”)

Table S1: Detailed Data of the Quality-of-Life Questionnaire

	Year	Questionnaire	Probiotic Group							Placebo Group							Statistics
			Baseline		End of Trial		Change values		Direction of change	Baseline		End of Trial		Change values		Direction of change	p - value
			mean	SD	mean	SD	mean	SD		mean	SD	mean	SD	mean	SD		
Abbas, Z., et al.	2014	IBS - QOL	NR	NR	NR	NR	NR	NR		NR	NR	NR	NR	NR	NR	NR	
Andresen, V. et al.	2020	SF - 12	NR	NR	NR	NR	5.82	NR	↑	NR	NR	NR	NR	4.06	NR	↑	0.038
Begtrup, L. M., et al.	2013	IBS - QOL	73.2	17.2	78.3	15.6	NR	NR	↑	73.4	18.6	78.0	16.4	NR	NR	↑	0.535
Choi, C. H., et al.	2011	IBS - QOL	70.9	12.8	80.0	12.3	15.4	16.4	↑	74.8	15.7	79.0	15.3	7.0	13.5	↑	0.03
Choi, C. H., et al.	2015	IBS - QOL															
Group 1			66.19	19.08	78.51	16.42	12.20	18.70	↑	61.31	18.49	75.32	16.52	14.10	16.52	↑	> 0.05
Group 2			62.30	18.64	75.74	17.28	13.30	18.19	↑								
Group 3			60.10	22.90	75.25	17.07	14.37	14.14	↑								
Group 4			65.15	20.75	76.23	18.65	10.68	14.15	↑								
Cremon, C. et al.	2018	SF-12	NR	NR	NR	NR	NR	NR	NR	NR	NR	NR	NR	NR	NR	NR	↔
Dapoigny, M. et al.	2012	GIQLI	63.9	7.8	NR	NR	NR	NR	NR	62.9	8.6	NR	NR	NR	NR	NR	↔
Drouault-Holowacz, S., et al.	2008	FDDQL<br>SF-36	NR	NR	NR	NR	NR	NR	NR	NR	NR	NR	NR	NR	NR	NR	↔
Francavilla, R. et al.	2019	IBS QOL	33.7	17	26.7	18.7	3.4	37.2	↓	31.5	19.3	24.5	16	4.5	19.2	↓	↔
Guglielmetti, S., et al.	2011	SF-12	physical composite 47.87<br>mental composite 45.53	NR	51.80<br>51.11	NR	3.99<br>5.78	NR	↑	47.33<br>47.01	NR	48.85<br>48.29	NR	1.08<br>1.58	NR	↑	0.0185<br>0.0083
Gupta, A. K. et al.	2021	QoL - Questionnaire	NR	NR	NR	NR	NR	NR	NR	NR	NR	NR	NR	NR	NR	NR	NR
Guyonnet, D., et al.	2007	FDDQL - discomfort (primary outcome)<br>Global score	49.7<br>62.3	18.8<br>15.8	62.0<br>68.8	18.4<br>14.7	12.2<br>6.5	16.2<br>11.2	↑	52.5<br>63.0	18.2<br>14.2	65.8<br>72.1	18.9<br>12.4	13.5<br>8.7	19.3<br>12.1	↑	↔
Kajander, K. et al.	2005	SF-36	72	NR	NR	NR	NR	NR	↔	64	NR	NR	NR	NR	NR	↔	↔
Ki Cha, B., et al.	2012	IBS-QOL	74.1	17.8	84.2	14.0	21.3	21.6	↑	80.6	13.3	87.2	12.9	9.0	21.5	↑	0.073
Kruis, W. et al.	2012	HRQL	111.6	22.2	NR	NR	22.8	28.7	↑	113.8	22.3	NR	NR	20.0	29.3	↑	↔
Lewis, E. et al.	2020	SF - 36	NR	NR	NR	NR	NR	NR	NR	NR	NR	NR	NR	NR	NR	NR	↔
		IBS - QOL	NR	NR	NR	NR	NR	NR	NR	NR	NR	NR	NR	NR	NR	NR	NR

Lorenzo-Zúñiga, V., et al.	2014	IBS-QOL	low dose group<br>50.6<br>high dose group<br>54.2	12.0<br>16.1	NR	NR	22<br>18	4<br>3	↑	54.6	18.5	NR	NR	9	3	↑	0.041<br>0.023
Lyra, A., et al.	2016	IBS-QOL	low dose group<br>63.9<br>high dose group<br>68.2	19.0<br>16.5	71.6<br>76.5	19.3<br>15.8	7.4<br>8.5	12.3<br>8.8	↑	66.4	17.5	73.2	19.0	7.0	12.3	↑	0.812<br>0.238
Majeed, M., et al.	2016	IBS-QOL	NR	NR	NR	NR	NR	NR	NR	NR	NR	NR	NR	NR	NR	NR	< 0.01
Majeed, M., et al.	2018	IBS-QOL	106.4	23.44	56.1	31.26	NR	NR	↓	102.6	21.11	84.1	34.67	NR	NR	↓	0.027
Martoni, C. J., et al.	2020	IBS-QOL	DDS-1 group<br>68.21<br>UABla-12 group<br>68.12	22.35<br>21.52	53.94<br>58.45	17.97<br>21.91	NR	NR	↓	66.17	18.26	59.68	19.40	NR	NR	↓	0.016<br>0.367
Niv, E., et al.	2005	IBS-QOL	4.56	0.73	NR	NR	NR	NR	NR	4.51	0.9	NR	NR	NR	NR	NR	↔
O'Mahony, L., et al.	2005	IBS-QOL	NR	NR	NR	NR	NR	NR	NR	NR	NR	NR	NR	NR	NR	NR	(0.05 level health worry for B. infantis)
Pinto-Sanchez, M. I., et al.	2017	SF-36	physical<br>45.0<br>physical function<br>78.3<br>role physical<br>61.1	10.1<br>24.2<br>43.1	49.9<br>94.5<br>90.8	8.8<br>9.5<br>35.9	NR	NR	NR	43.9<br>78.5<br>51.3	10.8<br>22.5<br>38.4	43.1<br>76.8<br>47.5	9.9<br>22.7<br>38.8	NR	NR	NR	< 0.05
Preston, K., et al.	2018	IBS-QOL (from V2 to V4)	NR	NR	NR	NR	24.01	22.39	↑ (some subgroups)	NR	NR	NR	NR	18.44	23.15	NR	improvement without significance calculation
Ringel-Kulka, T., et al.	2011	HR-QOL /IBS-QOL	76.5	16.4	NR	NR	NR	NR	↑	75.8	21.8	NR	NR	NR	NR	↑	↔
Simrén, M., et al.	2010	IBS-QOL	NR	NR	NR	NR	NR	NR	NR	NR	NR	NR	NR	NR	NR	NR	↔
Sisson, G., et al.	2014	IBS-QOL	53.2	20.25	53.4	20.52	6.9	13.21	↑	50.7	19.93	51.2	20.24	5.3	13.63	↑	0.47
Staudacher, H. et al.	2017	IBS-QOL	NR	NR	74.3	16.6	NR	NR	NR	NR	NR	68.6	20.7	NR	NR	NR	↔
		SF-36	NR	NR	NR	NR	NR	NR	NR	NR	NR	NR	NR	NR	NR	NR	NR
Stevenson, C., et al.	2014	IBS-QOL	49.95	21.43	44.42	23.96	NR	NR	↓	44.72	22.22	32.29	23.22	NR	NR	↓	↔
Sun, Y. Y., et al.	2018	IBS-QOL	81.28	18.58	88.51	14.40	7.232	14.06	↑	82.88	16.14	86.04	13.74	3.159	11.73	↑	0.032

Thijssen, A. Y., et al.	2016	QOL Physical composite score<br>QOL mental composite score	44<br>41	4<br>6	43<br>43	4<br>5	NR	NR	↔	43<br>39	5<br>7	43<br>42	4<br>6	NR	NR	↔	↔
Whorwell, P. J., et al.	2006	IBS-QOL	NR	NR	NR	NR	NR	NR	↔	NR	NR	NR	NR	NR	NR	↔	↔
Williams, E. A., et al.	2009	IBS-SSS-QOL	67.61	15.70	37.50	22.40	-29.65	NR	↑	61.88	11.64	47.41	17.58	-16.07	NR	↑	0.0068
Wong, R. K., et al.	2015	IBS-SSS-QOL	63.75	NR	52.5	NR	NR	NR	↑	59.55	NR	50.23	NR	NR	NR	↑	↔

IBS: irritable bowel syndrome; QOL: Quality of Life; ↑: improvement $p<0.05$; ↓: deterioration $p<0.05$; ↔: no significant differences between groups and no trend; NR: not reported; IBS – QOL: IBS – Quality Of Life Questionnaire; SF – 12: Short-Form 12 Items Health Survey; GIQLI: Quality Of Life Related to Gastrointestinal Disease; FDDQL: Functional Digestive Disorders Quality Of Life Questionnaire; SF-36: Short Form Health 36 Questionnaire; HRQL: Health Related Quality Of Life; IBS-SSS-QOL: IBS Symptom Severity Score Quality of Life

Table S2: Detailed Data of the Depression and Anxiety Score HADS

	**Year**	**Score**	**Probiotic Group**							**Placebo Group**							**Statistics**	
			Baseline		End of Trial		Change values		Direction of change	Baseline		End of Trial		Change values		Direction of change	p - value	
			mean	standard deviation	mean	standard deviation	mean	standard deviation		mean	standard deviation	mean	standard deviation	mean	standard deviation			
Cremon, C. et al.	2018	Depression score	NR	NR	NR	NR	- 0.71	NR	↓	NR	NR	NR	NR	0.08	NR	↓	0.314	↔
		Anxiety score	NR	NR	NR	NR	NR	NR	negative	NR	NR	NR	NR	NR	NR	negative	>0.05	↔
Dapoigny, M. et al.	2012	Total	16.3	6.5	NR	NR	NR	NR	NR	16.5	6.4	NR	NR	NR	NR	NR	NR	↔
Lewis, E. et al.	2020	Total	NR	NR	NR	NR	NR	NR	↓	NR	NR	NR	NR	NR	NR	↓	NR	↔
Lyra, A., et al.	2016	Depression score	NR	NR	NR	NR	NR	NR	NR	NR	NR	NR	NR	NR	NR	NR	NR	↔
		Anxiety score	NR	NR	NR	NR	NR	NR	NR	NR	NR	NR	NR	NR	NR	NR	NR	↔
Pinto-Sanchez, M. I., et al.	2017	Depression score	7.6	3.7	3.9	3.1	NR	NR	↓	5.2	3.0	4.5	3.1	NR	NR	↓	0.049	↑
		Anxiety score	10.2	3.2	6.5	3.8	NR	NR	↓	9.3	2.6	7.1	3.9	NR	NR	↓	NR	↔
Simrén, M., et al.	2010	Depression score	3.6	3.1	3	3	NR	NR	NR	4.0	3.1	3	3	NR	NR	NR	> 0.05	↔
		Anxiety score	6.5	3.7	6	4	NR	NR	NR	7.9	2.9	6	3	NR	NR	NR	0.001 for placebo	↔
Whorwell, P. J., et al.	2006	Depression score	NR	NR	NR	NR	NR	NR	NR	NR	NR	NR	NR	NR	NR	NR	NR	↔
		Anxiety score	NR	NR	NR	NR	NR	NR	NR	NR	NR	NR	NR	NR	NR	NR	NR	↔
Wong, R. K., et al.	2015	Depression score	3.52	0.43	2.75	NR	NR	NR	↓	5.5	0.84	4.1	NR	NR	NR	↓	NR	↔
		Anxiety score	5.9	0.86	5.5	NR	NR	NR	↓	8.63	0.76	8.09	NR	NR	NR	↓	NR	↔

↑: improvement $p<0.05$; ↓: deterioration $p<0.05$; ↔: no significant differences between groups and no trend; NR: not reported

Table S3: Bacterial Species

	Name of the product or manufacturer	Species
Abbas, Z., et al. (2014)	provided by Biocodex (Beauvais, France)	Saccharomyces boulardii
Andresen, V. et al. (2020)	sponsored by C Hedenkamp, Hövelhof	Bifidobacterium bifidum HI-MIMBb75
Begtrup, L. M., et al. (2013)	Danish-Swedish cooperative dairy company Arla Foods	Lactobacillus paracasei ssp paracasei F19<br>Lactobacillus acidophilus La5<br>Bifidobacterium Bb12
Choi, C. H., et al. (2011)	Bioflor (Kuhnil, Seoul, Republic of Korea)	Saccharomyces boulardii
Choi, C. H., et al. (2015)	Medilac (Hanmi Pharma Korea Inc.)	Bacillus subtilis<br>Streptococcus faecium
Cremon, C. et al. (2018)	Enterolactis plus (Sofar S.p.A., Trezzano Rosa, Milan, Italy)	Lactobacillus paracasei CNCM I1572
Dapoigny, M. et al. (2012)	Supported by Laboratoires Lyocentre	Lactobacillus casei variety rhamnosus
Drouault-Holowacz, S., et al. (2008)	sponsored by PiLeJe	Bifidobacterium longum LA 101 (29%)<br>Lb. Acidophilus LA 102 (29%)<br>Lactococcus lactis LA 103 (29%)<br>Streptococcus thermophilus LA 104 (13%)
Francavilla, R. et al. (2019)	provided free of charge by Probioresearch	Lactobacillus casei LMG 101/37 P-17504 (5×10^9 CFU/sachet)<br>Lactobacillus plantarum CECT 4528 (5×10^9 CFU/sachet)<br>Bifidobacterium animalis subsp. lactis Bi1 LMG P-17502 (10×10^9 CFU/sachet)<br>Bifidobacterium breve Bbr8 LMG P-17501 (10×10^9 CFU/sachet)<br>Bifidobacterium breve Bl10 LMG P-17500 (10×10^9 CFU/sachet)
Guglielmetti, S., et al. (2011)	funded by Naturwohl Pharma GmbH	Bifidobacterium bifidum MIMBb75
Gupta, A. K. et al. (2021)	supplied by Advanced Enzyme Technologies Ltd.	Bacillus coagulans LBSC [DSM17654]

Guyonnet, D., et al. (2007)	provided by Danone Research	Bifidobacterium animalis DN-173 010: 1.25 x 10^10 CFU<br>S. thermophilus: 1.2 x 10^9 CFU<br>L.bulgaricus: 1.2 x 10^9 CFU
Kajander, K. et al. (2005)	from Valio Ltd	Lactobacillus rhamnosus LC705<br>Bifidobacterium breve Bb99<br>Propionibacterium freudenreichii ssp. shermanii JS
Ki Cha, B., et al. (2012)	Duolac7 (Cell Biotech, Co., Ltd)	Lactobacillus acidophilus(KCTC 11906BP)<br>Lactobacillus plantarum (KCTC11867BP)<br>Lactobacillus rhamnosus (KCTC 11868BP) Bifidobacterium breve (KCTC 11858BP)<br>Bifidobacterium lactis (KCTC11903BP) Bifidobacterium longum (KCTC 11860BP)<br>Streptococcus thermophilus (KCTC 11870BP)
Kruis, W. et al. (2012)	MUTAFLOR manufactured by Ardeypharm GmbH	Escherichia coli Nissle 1917
Lewis, E. et al. (2020)	manufactured by Lallemand Health Solutions (LHS)	Bifidobacterium longum R0175 (Lot Numbers: NH131210-1VB and NH151104-ICP)<br>or<br>Lactobacillus paracasei HA-196 (Lot Numbers: NH131217-1VB and NH151106-ICP)
Lorenzo-Zúñiga, V., et al. (2014)	produced by ABbiotics	2 Lactobacillus plantarum: CECT7484 and CECT7485)<br>Pediococcus acidilactici (CECT7483)
Lyra, A., et al. (2016)	supplied by Danisco USA	Lactobacillus acidophilus NCFM (ATCC 700396)
Majeed, M., et al. (2016)	supplied by Sabinsa Corporation, Utah, USA	Bacillus coagulans MTCC 5856
Majeed, M., et al. (2018)	LACTOSPORE STABLE PROBIOTIC of Sabinsa Corporation	Bacillus coagulans MTCC 5856
Martoni, C. J., et al. (2020)	UAS Laboratories LLC	Lactobacillus acidophilus DDS®-1<br>Bifidobacterium animalis subsp. lactis UABla-12™
Niv, E., et al. (2005)	BioGaia AB	Lactobacillus reuteri ATCC 5573
O'Mahony, L., et al. (2005)	Man/Rogosa/Sharp broth (Oxoid, Basingstoke)	Lactobacillus salivarius subspecies salivarius UCC4331<br>Bifidobacterium infantis 35624
Pinto-Sanchez, M. I., et al. (2017)	Funding provided by Nestlé SA	Bifidobacterium longum NCC3001

Preston, K., et al. (2018)	Bio-K Plus International Inc. provided funding and IP for the study	Lactobacillus acidophilus CL1285<br>Lactobacillus casei LBC80R<br>Lactobacillus rhamnosus CLR2
Ringel-Kulka, T., et al. (2011)	supported by K23 DK075621, RR00046, and Danisco USA Inc.	Lactobacillus acidophilus NCFM (L-NCFM)<br>Bifidobacterium lactis Bi-07 (B-LBi07)
Simrén, M., et al. (2010)	supported by an unrestricted grant from Arla Foods Innovation, Swedish Medical Research Council (grant 13409), and by the Faculty of Medicine, University of Göteborg	Lactobacillus paracasei, ssp. paracasei F19<br>Lactobacillus acidophilus La5<br>Bifidobacterium lactis Bb12
Sisson, G., et al. (2014)	Symprove from Symprove Ltd, Farnham	Lactobacillus rhamnosus NCIMB 30174<br>Lactobacillus plantarum NCIMB 30173<br>Lactobacillus acidophilus NCIMB 30175<br>Enterococcus faecium NCIMB 30176
Staudacher, H. et al. (2017)	now exclusively available in Europe under the trademark Vivomixx and in the United States under the trademark Visbiome	Streptococcus thermophilus DSM 24731<br>Bifidobacterium breve DSM 24732<br>B. longum DSM 24736<br>B. infantis DSM 24737<br>Lactobacillus acidophilus DSM 24735<br>L. plantarum DSM 24730<br>L. paracasei DSM 24733<br>L. delbrueckii subsp. bulgaricus DSM 24734
Stevenson, C., et al. (2014)	Ferlot Manufacturing and Packaging (PTY) Ltd	L. plantarum 299 v
Sun, Y. Y., et al. (2018)	ATaiNing, Qingdao Eastsea Pharmaceutical Co., Ltd.	Clostridium butyricum (CB)
Thijssen, A. Y., et al. (2016)	provided by Yakult Europe	Lactobacillus casei Shirota
Whorwell, P. J., et al. (2006)	prepared by The Procter & Gamble Company	Bifidobacterium infantis 35624

Williams, E. A., et al. (2009)	prepared by Cultech Ltd, Port Talbot	Lactobacillus acidophilus CUL-60 (NCIMB 30157)<br>Lactobacillus acidophilus CUL-21(NCIMB 30156)<br>Bifidobacterium bifidum CUL-20 (NCIMB 30153)<br>Bifidobacterium lactis CUL-34 (NCIMB 30172)
Wong, R. K., et al. (2015)	VSL#3 supplied by VSL Pharmaceuticals, Inc.	Bifidobacterium (B. longum, B. infantis and B. breve)<br>Lactobacillus (L. acidophilus, L. casei, L. delbrueckii ssp. bulgaricus and L. plantarum)<br>Streptococcus salivarius ssp. thermophilus

Table S4: Questionnaires applied by the included studies

Abbreviation	Deskription
APS-NRS	Abdominal Pain Severity – Numeric Rating Scale
AR	Adequat Relief
Brimingham IBS score	/
BSFS	Bristol Stool Form scale
CES-D	Centre for Epidemiological Studies–Depression Scale
CFU	Colony Forming Unit
CGI-I	Clinical Global Impression-Improvement Rating Scale
CGI-S	Clinical Global Impression Severity Rating Scale
CSBM	Complete Spontaneous Bowel Movement
CSFBD	Cognitive scale for functional bowl disordes
DSFQ	Digestive Symptom Frequency Questionnaire
ESS	Epworth sleepiness scale
FDDQL	Functional digestive disorders quality of life questionnaire
GI-DQ	Gastrointestinal Discomfort Questionnaire
GIQLI	Quality of life related to gastrointestinal disease
GSRS-IBS	Gastrointestinal Symptom Rating Scale specific for IBS
HAD(S)	Hospital Anxiety and Depression Scale
HAM-D	Hamilton Rating Scale for Depression
HrQOL	Health Related Quality of life
IBS-QOL	Irritable Bowel Syndrome Quality of Life
IBS-SSS	Symptom Severity Score
IMPSS	Integrative Medicine Patient Satisfaction Scale
MADRS	Montgomery-Asberg Depression Rating Scale
mESS	Modified Epworth Sleepiness Scale
MSS	Mean Symptom Score
PSQI	Pittsburgh sleep quality Index
RMBPC	Dementia – Revised Memory and Behaviour Problem Checklist
SBDQ	standardized bowel disease questionnaire
SBM	Spontaneous Bowel Movement
SCBMs	Spontaneous Complete Bowel Movements
SF-12	Short-Form 12 Items Health Survey
SF-36	Short Form Health 36 Questionnaire
SGA	Subjects Global Assessment
STAI	State and Trait Anxiety Inventory
VAS	Visual Analog Scale
VSI	Visceral Sensitivity Index
